# Recent advances in piezoelectric hydrogels for osteoarthritis therapy: material design, signal transduction, and clinical translation

**DOI:** 10.3389/fbioe.2026.1938136

**Published:** 2026-08-26

**Authors:** Senbo Zhu, Yu Tong

**Affiliations:** 1 Center for Rehabilitation Medicine, Department of Orthopedics, Zhejiang Provincial People’s Hospital (Affiliated People’s Hospital), Hangzhou Medical College, Hangzhou, Zhejiang, China; 2 Department of Sports Medicine, Zhejiang Provincial People’s Hospital (Affiliated People’s Hospital), Hangzhou Medical College, Hangzhou, Zhejiang, China

**Keywords:** cartilage regeneration, immunomodulation, osteoarthritis, piezoelectric hydrogel, signal transduction

## Abstract

With no disease-modifying therapy available, osteoarthritis (OA) remains a primary cause of disability worldwide. However, between 2023 and 2026, rapid advances have been made in the development of piezoelectric hydrogels, a technology that transduces joint motion into endogenous electrical signals, thereby restoring the electromechanical microenvironment of degenerated cartilage. This short review provides a critical synthesis of these developments through a four-dimensional framework encompassing material design, signal transduction, therapeutic efficacy, and translational validation. Three material design paradigms are analysed: inorganic nanofiller composites, organic piezoelectric polymers, and natural biopolymer hydrogels. This analysis reveals that the central unresolved controversy is the fundamental trade-off between piezoelectric output and biointegration. The Ca2+–TRPV4/Piezo1–MAPK/ERK–SOX9 signalling cascade is the core anabolic pathway, complemented by M1-to-M2 macrophage polarisation and NLRP3 inflammasome inhibition. Although preclinical evidence from small-animal OA models has demonstrated consistent International Cartilage Repair Society (ICRS) score improvements, three critical research gaps persist: the absence of validation in spontaneous or metabolic OA models, the lack of long-term efficacy data beyond 12 weeks, and inadequate characterisation of the decline in piezoelectric performance under physiological conditions. This review highlights emerging synergistic strategies combining piezoelectric hydrogels with ultrasound or magnetic field stimulation to overcome motion-dependent output instability, and identifies adaptive piezoelectric design, multimodal physical stimulation integration, and functional reconstruction–centred evaluation as key priorities for clinical translation.

## 1 Introduction

Osteoarthritis (OA) affects over 500 million individuals worldwide; however, the interventions that are currently available, including microfracture, autologous chondrocyte implantation, and pharmacological management, provide only symptomatic relief without arresting disease progression or restoring native cartilage architecture (Zhang et al., 2025; Vinikoor et al., 2023). A central insight driving recent research and development is that articular cartilage is an electromechanically active tissue. Generated through the oriented collagen fibril network under mechanical deformation, endogenous piezoelectricity maintains chondrocyte homeostasis (Alvarez-Lorenzo et al., 2023; Chen et al., 2024), and its degeneration during OA represents both a pathogenic mechanism and a therapeutic target (Das et al., 2024).

Piezoelectric hydrogels, which are mechanically compliant biomaterials that transduce joint motion into localised electrical fields, have emerged as a compelling strategy for restoring this lost electromechanical signalling. Between 2023 and 2026, the field has evolved from *in vitro* demonstrations of chondrogenic differentiation to establishing complete mechanistic chains that link material-level piezoelectric output to ion channel activation, intracellular signalling cascades, mitochondrial reprogramming, and functional cartilage repair in preclinical models (Liu S. et al., 2024; Zheng et al., 2026).

This short review provides a critical synthesis organised around a four-dimensional framework comprising material design, signal transduction, therapeutic efficacy, and translational validation. It highlights unresolved controversies, identifies critical research gaps, and outlines priorities for clinical translation.

## 2 Material design paradigms

**FIGURE 1:**
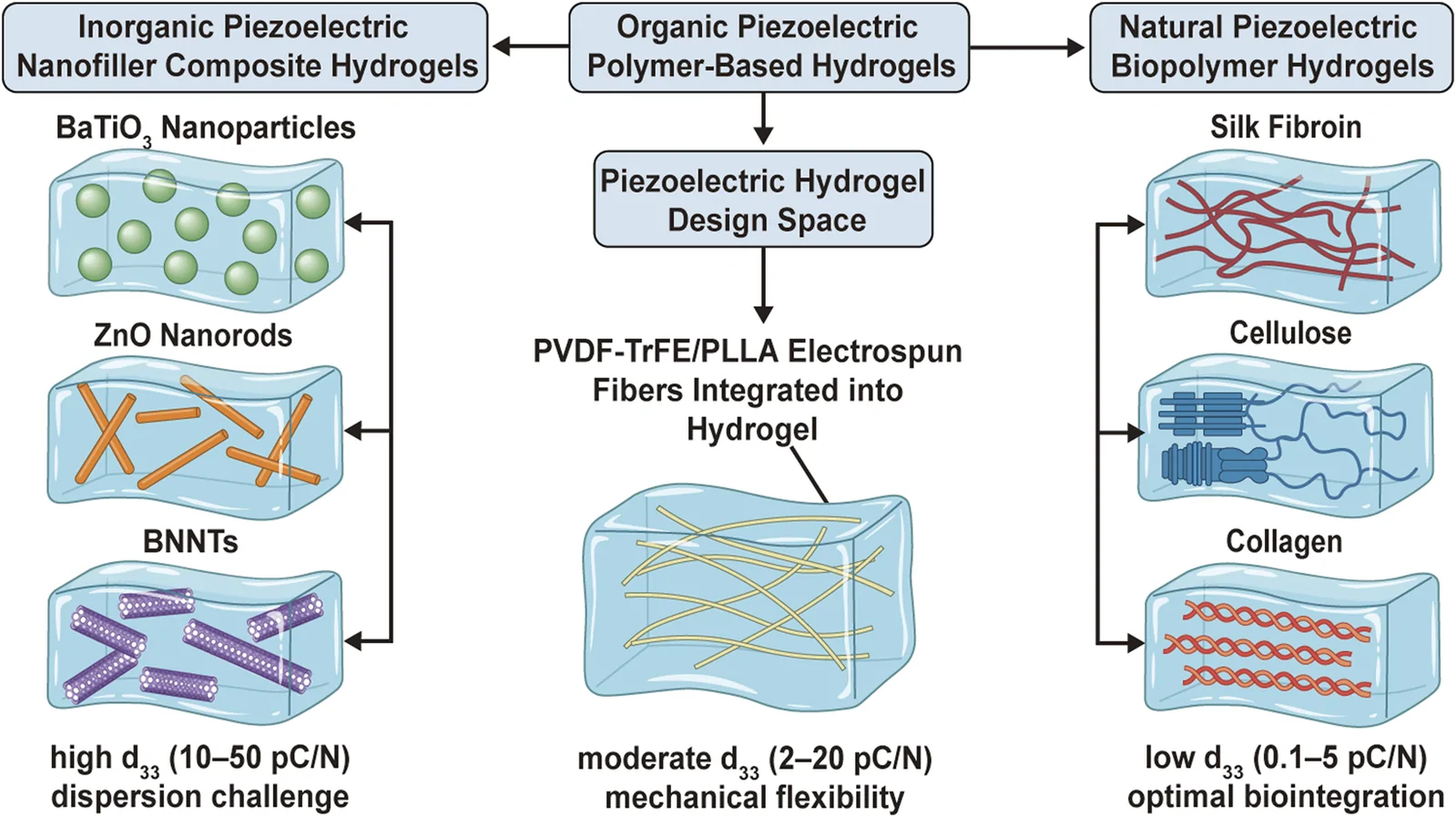
Three complementary material design paradigms for piezeoelectric hydrogels.

The design of piezoelectric hydrogels for OA therapy must balance three competing requirements: sufficient piezoelectric output, mechanical properties mimicking articular cartilage (0.5–2 MPa compressive modulus), and injectability for minimally invasive delivery. Surface potentials of 10–200 mV are typically cited for bulk hydrogel constructs under physiological loading (Alvarez-Lorenzo et al., 2023); however, effective stimulation at the cellular level may involve significantly divergent local electrical fields. Single-nanoparticle and nanomotor systems, for instance, can elicit therapeutic responses through highly localised potentials at the nanoparticle-cell interface even when macroscopic measurements fall below this range (Wang et al., 2026). Consequently, the appropriate performance metric is contingent upon the material architecture. Materials developed over the past 3 years fall into three complementary classes (Figure 1), each representing a distinct design philosophy.

### 2.1 Inorganic nanofiller composite hydrogels

Barium titanate (BaTiO_3_) nanoparticles, zinc oxide (ZnO) nanorods, and boron nitride nanotubes offer the highest piezoelectric coefficients among the three classes. While bulk BaTiO_3_ ceramics exhibit d_33_ values of 100–500 pC/N, effective coefficients in hydrogel composites are typically 10–100 pC/N due to polymer matrix dilution, inefficient stress transfer at the particle-matrix interface, and random particle orientation (Li B. et al., 2024). Several strategies have addressed the primary challenge: nanoparticle aggregation, which limits uniform electromechanical performance. Deep eutectic solvent-assisted frontal polymerisation simultaneously solubilises monomers and stabilises nanoparticle dispersions (Li B. et al., 2024), while coating nanoparticles with polydopamine improves matrix-particle interfacial adhesion through increased β-phase content and reduced interfacial defects. More broadly, surface functionalisation of nanoparticles with biocompatible ligands and the use of polymeric matrices with tailored chemistries are widely adopted approaches for improving dispersion and minimising interfacial energy losses (Vannozzi et al., 2025). However, a persistent trade-off remains: increasing filler loading raises d33 but increases the risk of nanoparticle leaching, ROS generation, and chondrocyte toxicity (Chen et al., 2024). This dose-toxicity relationship merits dedicated attention. Nanoparticle release into the synovial fluid may produce systemic effects through lymphatic drainage; furthermore, the therapeutic window defined by the minimum effective piezoelectric dose and the maximum tolerated nanoparticle concentration has not been systematically established for any OA-targeted system.

### 2.2 Organic piezoelectric polymer–based hydrogels

Poly(vinylidene fluoride-co-trifluoroethylene) (PVDF-TrFE) and poly(L-lactide) (PLLA) provide superior mechanical flexibility and controlled degradability, with d_33_ values of 2–20 pC/N (Fan et al., 2024). The key innovation of this type of hydrogel has been electrospinning–hydrogel hybridisation. Electrospun piezoelectric nanofibers, with polymer chains aligned and β-phase crystallised through mechanical stretching, are embedded within a hydrophilic hydrogel matrix for nutrient transport and cell infiltration (Li S. et al., 2025). By tuning the compressive moduli of these systems across 2–10 MPa, they can be made well suited for superficial cartilage defects. The trade-off is their inherently lower piezoelectric output than that of inorganic composites, and the long-term stability of their electroactive phase under cyclic loading and enzymatic degradation has not been systematically assessed (Li Y. et al., 2024).

### 2.3 Natural piezoelectric biopolymer hydrogels

The ordered crystalline domains of silk fibroin, cellulose nanocrystals, and collagen confer intrinsic piezoelectricity. Though these materials have modest d_33_ values of 0.1–5 pC/N, their biointegration is unmatched due to natural enzymatic degradation and characteristic immunocompatibility (Fan et al., 2024; Xu et al., 2024). Silk fibroin β-sheet crystalline domains confer piezoelectricity through shear-induced alignment, and their degradation products include metabolizable amino acids (Yue et al., 2023). The central challenge lies in the gap between intrinsic output and therapeutic requirements. While hybridisation with synthetic piezoelectric materials such as silk fibroin–PVDF composites represents a pragmatic compromise (Mo et al., 2026), chemical modification to boost output often weakens the biointegration that defines this class (Vijayakanth et al., 2023). As comprehensively reviewed by Vannozzi et al., fabrication approaches including electrospinning, template-assisted alignment, and thermal poling can enhance the piezoelectric response of organic and natural materials without necessarily sacrificing biocompatibility. Consequently, the selection of fabrication techniques should be evaluated alongside chemical modification as a complementary strategy for output optimization (Vannozzi et al., 2025).

This inverse relationship between piezoelectric output and biointegration constitutes the central materials science–related controversy in the field. While proponents of inorganic composites view output magnitude as the dominant determinant of therapeutic efficacy, advocates of biopolymer-based systems contend that long-term biointegration and degradation safety outweigh peak electrical performance. The emergence of hybrid architectures combining elements from multiple classes indicates that this controversy may be resolved not by selecting one paradigm but by rationally integrating complementary properties. By “optimum balance” we mean the trade-off among four properties that are inversely coupled across the three material classes: piezoelectric output (d_33_ and resulting surface potential), mechanical modulus matching with host cartilage, degradation kinetics, and immunocompatibility. The right balance depends on the OA phenotype and the location of the defect. Early-stage, post-traumatic OA with a localised lesion in a load-bearing region such as the femoral condyle calls for mechanical robustness and high piezoelectric output, favouring inorganic-organic hybrid systems. Advanced, inflammation-driven OA with widespread cartilage erosion and synovitis, by contrast, may be better served by the strong immunomodulation and enzymatic degradability of natural biopolymer-based systems, even if this comes at the cost of lower electrical output. Superficial zone defects accessible by injection can be addressed by any of the three paradigms, while deep osteochondral defects that require scaffold integration with subchondral bone demand higher compressive moduli and deeper signal penetration and therefore tilt the balance toward organic polymer or hybrid systems.

## 3 Biological mechanisms: signal transduction and immunomodulation

**FIGURE 2:**
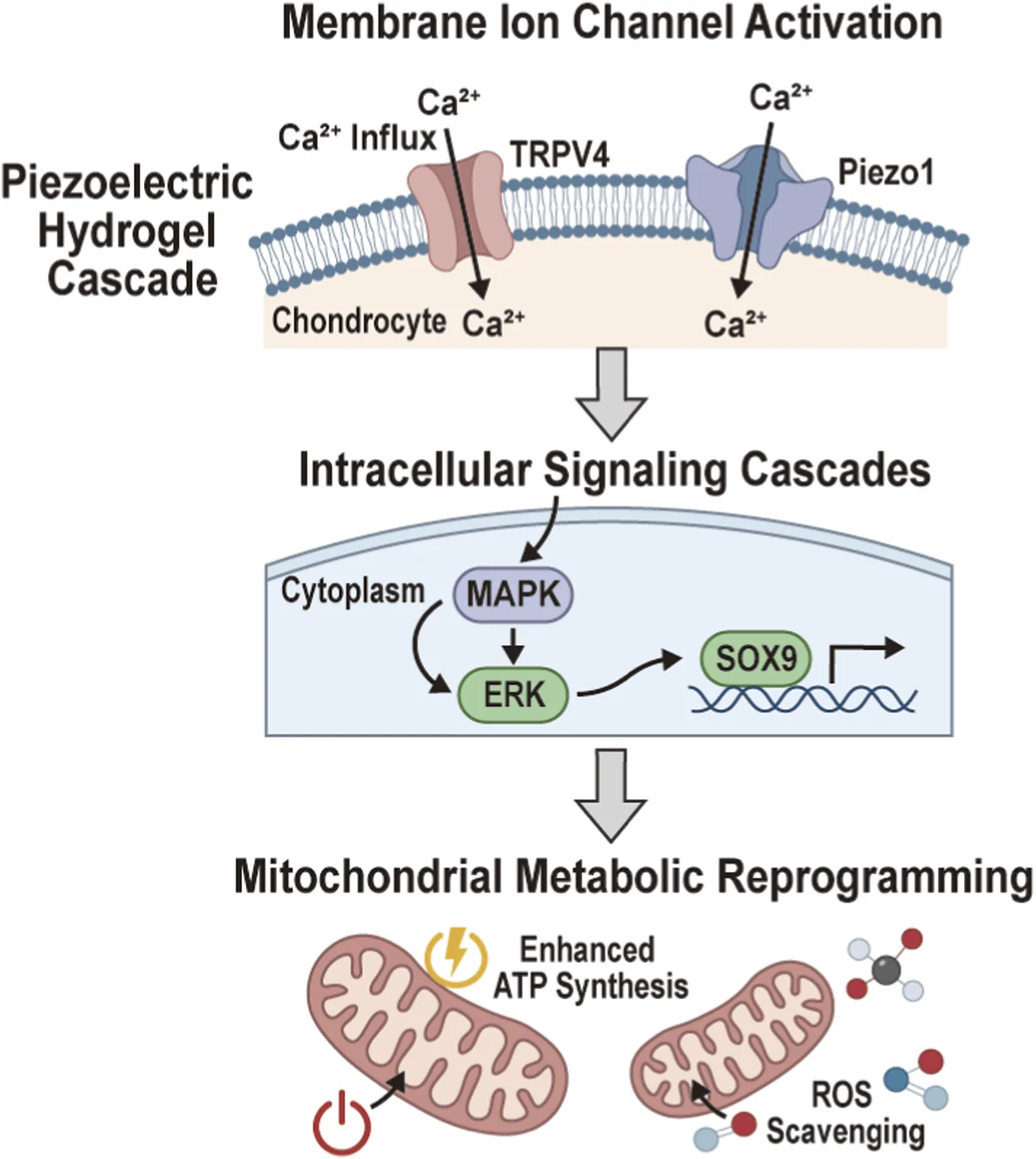
A three-stage signal transduction cascade linking piezoelectric stimulation to chondrocyte anabolic metabolism.

Understanding how chondrocytes detect and transduce piezoelectric signals is crucial for rational material design. Over the past 3 years, a coherent three-tier cascade has been elucidated (Figure 2), operating alongside parallel immunomodulatory mechanisms.

### 3.1 Membrane-to-mitochondria signal transduction

Piezoelectric surface potentials (10–200 mV) activate voltage-gated calcium channels the mechanosensitive channels TRPV4 and Piezo1 (Zhou et al., 2023; Li J. et al., 2024). Notably, single-nanoparticle or nanomotor systems may produce highly localized electric fields at the cell-material interface that are not adequately captured by macroscopic surface potential measurements; the effective stimulating field experienced by an adjacent chondrocyte may therefore differ substantially from the bulk output value. Han et al. (Han et al., 2025) showed that injectable force–electric hydrogel microspheres induce Ca^2+^ influx through synergistic activation of TRPV4 and Piezo1. Piezo1-mediated Ca^2+^ entry then potentiates TRPV4 opening via calcium-induced calcium release, thereby creating a feed-forward amplification loop. The signal waveform differentially influences channel activation: while pulsatile signals from piezoelectric ceramics preferentially activate voltage-gated calcium channels, sustained potentials from polymer-based systems favour TRPV4 opening (Gao et al., 2026).

Downstream, the Ca^2+^ influx activates the MAPK/ERK pathway as the dominant chondrogenic route: ERK1/2 phosphorylation promotes SOX9 nuclear translocation, thereby co-ordinately upregulating SOX9, ACAN, and COL2A1 (Zhou et al., 2023; Ricotti et al., 2024). A critical biphasic dose–response relationship has been identified: low-to-moderate stimulation (10–100 mV) activates anabolic pathways, whereas intensities exceeding 150 mV trigger hypertrophic markers, including COL10A1 and MMP13 (Li J. et al., 2024).

The discovery that piezoelectric stimulation extends to mitochondrial reprogramming represents one of the most mechanistically significant developments in recent years. Zheng et al. (Zheng et al., 2026) demonstrated that an ultrasound-activatable piezoelectric hydrogel (CMB Gel) induces mtDNA demethylation via the mTOR/GATD3A axis, thereby preferring oxidative phosphorylation to glycolysis while upregulating SOD2 and GPx antioxidant enzymes. Gao et al. (Gao et al., 2026) confirmed that a piezo-mimetic ionic hydrogel restores mitochondrial membrane potential while reducing ROS under inflammatory conditions, establishing mitochondrial reprogramming as a general downstream consequence of piezoelectric signalling rather than a material-specific artefact. These findings suggest a positive feedback loop comprising enhanced ATP synthesis, ROS scavenging, anabolic metabolism, matrix synthesis, improved mechanical function, and sustained piezoelectric output, thereby providing a plausible explanation for how a single implantation may provide sustained therapeutic benefits over weeks to months.

### 3.2 Immune microenvironment modulation

OA is increasingly considered a whole-joint disease wherein synovial inflammation drives cartilage degradation. Piezoelectric stimulation reverses the pathological M1 macrophage bias via two convergent pathways: NF-κB inhibition, which suppresses M1 programs, and STAT6 activation, which drives M2-associated Arg1, CD206, and IL-10 (Liu X. et al., 2024; Wu et al., 2023). The M2/M1 ratio shift (two-to four-fold *in vivo*) is similar to that of IL-4-loaded systems but remains stimulus-responsive rather than reliant on preloaded cytokine depots (Li et al., 2025a).

In parallel with macrophage modulation, piezoelectric hydrogels suppress inflammation through piezocatalytic ROS scavenging. Mechanical deformation generates electron–hole pairs that convert superoxide and hydrogen peroxide into water and oxygen, thus reducing mitochondrial ROS below the threshold required for NLRP3 inflammasome assembly (Yao et al., 2025; Song et al., 2025). NLRP3 inflammasome inhibition disrupts the feed-forward cycle of IL-1β/IL-18-driven cartilage degradation. Notably, the immunomodulatory reach may extend to the neuroimmune axis. Recent evidence indicates that piezoelectric analgesic microspheres modulate peripheral nociceptor desensitisation suggesting broader effects that remain largely unexplored (Li et al., 2025b).

## 4 Preclinical evidence and translational gaps

Translation from benchtop to clinic requires rigorous preclinical validation. Although the data accumulated since 2023 are encouraging, they reveal critical gaps that must be addressed.

### 4.1 Evidence of efficacy from SmallAnimal models

Vinikoor et al. (2023) were the first to demonstrate that an injectable, biodegradable piezoelectric hydrogel—electrospun PLLA nanofibers in a collagen matrix—improved cartilage repair in a rat model of destabilisation of the medial meniscus (DMM), with an approximately 1.5-fold ICRS score improvement specifically due to the piezoelectric function. Wang et al. (2026) advanced the concept with piezoelectric BaTiO_3_ nanomotors that actively navigate to irregular defect surfaces under ultrasound, achieving an approximately two-fold ICRS score improvement in a rat Anterior Cruciate Ligament Transection (ACLT) model. Tschon et al. (2026) extended validation to the rabbit ACLT model using a piezoelectric nanocomposite hydrogel combined with low-intensity pulsed ultrasound, achieving an approximately two-fold ICRS score improvement. Li et al. (2024) demonstrated the first biodegradable piezoelectric polymer (PHBV) that maintained output for 8–12 weeks *in vivo*, sufficient to support full chondrogenic differentiation and achieve an approximately 2.5-fold ICRS score improvement. Gao et al. (2026) provided complementary evidence using a piezo-mimetic ionic hydrogel, achieving a nearly two-fold ICRS score improvement in a rat DMM model. These studies are summarised in Table 1.

**TABLE 1:** Summary of preclinical studies of piezoelectric hydrogels in OA animal models (2023–2026).

Material system	Animal model	Model type	Duration	ICRS improvement	Ref
PLLA nanofiber–collagen hydrogel	Rat (DMM)	Post-traumatic	8 weeks	∼1.5-fold	(Vinikoor et al., 2023)
BaTiO3 nanomotors + US	Rat (ACLT)	Post-traumatic	12 weeks	∼2-fold	(Wang et al., 2026)
Piezoelectric nanocomposite + LIPUS	Rabbit (ACLT)	Post-traumatic	12 weeks	∼2-fold	(Tschon et al., 2026)
PHBV piezoelectric scaffold	Rat (osteochondral)	Defect	12 weeks	∼2.5-fold	(Li Y. et al., 2024)
Piezo-mimetic ionic hydrogel	Rat (DMM)	Post-traumatic	8 weeks	∼2-fold	(Gao et al., 2026)

### 4.2 Critical research gaps

Three research gaps warrant immediate attention. First, there is a near-total absence of validation in spontaneous or metabolic OA models. Studies have invariably used surgically induced post-traumatic OA models, which recapitulate mechanical injury but not the systemic drivers such as obesity, diabetes, and aging that underlying most clinical OA. Str/ort mouse and Dunkin–Hartley guinea pig models remain untested. Second, long-term follow-up is lacking. Most studies have evaluated outcomes over 4–12 weeks, which is sufficient to demonstrate matrix synthesis but insufficient to assess durability under continued loading, late-stage hypertrophy, or calcification. Only one study has characterised piezoelectric output beyond 12 weeks; for most systems, the half-life of output under physiological conditions is estimated to be 4–8 weeks, which may not be sufficient for the months-long cartilage maturation timeline (Li Y. et al., 2024). Third, regarding biomechanical scaling, rat joint contact stresses (∼0.5 MPa) and cartilage thickness (<0.5 mm) differ fundamentally from those of humans (5–10 MPa and 2–4 mm, respectively). Finite-element modelling suggests an effective electrical penetration depth of <1 mm, implying that only superficial zone chondrocytes in human cartilage would receive suprathreshold stimulation (Lin Z. et al., 2025). Validation in large-animal models (e.g., porcine and caprine) with joint mechanics more similar to those of humans is essential before first-in-human trials.

## 5 Synergistic strategies and future directions

A fundamental limitation of joint motion–dependent piezoelectric stimulation is that patients with OA who experience severe pain or post-surgical immobilisation may not generate sufficient loading. This limitation has motivated the development of synergistic strategies that combine piezoelectric hydrogels with externally applied physical stimulation.

Ultrasound-driven systems have emerged as the most mature approach. Lin et al. (2025) demonstrated an ultrasound-triggered injectable hyaluronic acid–based piezoelectric hydrogel that decouples therapeutic activation from patient activity while providing viscosupplementation, a clinically established OA treatment. Zheng et al. (2026) achieved multimodal therapy using chondrocyte membrane–camouflaged BaTiO_3_ nanoparticles. Ultrasound activation simultaneously opens the blood–cartilage barrier, drives piezocatalytic ROS scavenging, and induces mitochondrial epigenetic reprogramming. Pulsed ultrasound protocols (e.g., 100 and 900 m on and off) minimise thermal effects while maintaining effective piezoelectric activation (Yang et al., 2023). A key limitation of these studies, however, is that ultrasound simultaneously induces mechanical deformation (acoustic radiation force and cavitation) and electrical signals generated via the piezoelectric effect, which complicates the isolation of the biological contribution of the piezoelectric effect from that of mechanical stimulation alone. Future studies that incorporate purely mechanical controls—such as non-piezoelectric hydrogels with identical acoustic impedance—would strengthen causal inference.

Self-powered systems that harness natural joint biomechanics constitute an alternative approach. Lin Z. et al. (2025) constructed a hierarchically structured piezoelectric scaffold in which macroscale architecture, microscale fibre alignment, and nanoscale domain orientation are co-optimised, thereby maximizing motion-to-electricity transduction across the physiological loading spectrum. The appeal lies in its autonomy: it requires no external equipment, imposes no compliance burden, and leverages a physiological feedback loop in which more movement generates more stimulation. However, it remains limited by its dependence on patient mobility during early rehabilitation.

Magnetoelectric coupling, in which an alternating magnetic field induces strain in a magnetostrictive material transduced into electrical output, enables deep-tissue wireless stimulation with negligible attenuation through bone and soft tissue. Although it has been used in bone tissue engineering, its application to OA cartilage repair remains exploratory because conventional magnetostrictive materials are brittle.

The convergence of these strategies highlights three priorities. Adaptive piezoelectric hydrogels capable of sensing MMP activity, pH, or ROS levels and dynamically adjusting output may address OA heterogeneity across patient populations and disease stages. Multimodal physical stimulation integrating ultrasound, magnetic, and mechanical modalities within a single implant may help overcome the depth and mobility limitations of current systems. Functional reconstruction–centred evaluation must replace histological scoring as the primary endpoint, placing focus on joint-level mechanical function, pain-free range of motion, and long-term durability. Achieving these priorities warrants close collaboration among materials scientists, biophysicists, and orthopaedic surgeons, alongside standardised evaluation protocols enabling cross-study comparison.

## 6 Conclusion

The years 2023–2026 have seen research on piezoelectric hydrogels for OA therapy evolve from phenomenological observation to mechanistic understanding. With the Ca^2+^–TRPV4/Piezo1–MAPK/ERK–SOX9 cascade established as the core anabolic pathway, the discovery of mitochondrial epigenetic reprogramming has added a new dimension to sustained therapeutic efficacy, while the immunomodulatory landscape comprising M1-to-M2 polarisation, NLRP3 inflammasome inhibition, and neuroimmune modulation has been systematically mapped. Two critical caveats remain. First, most mechanistic studies have focused primarily on chondrocytes. Consequently, whether the full signalling cascade is conserved in other OA-relevant cell types—such as synovial fibroblasts, osteoblasts, and peripheral nociceptors—remains to be systematically examined. Second, all mechanistic evidence to date is derived from rodent and rabbit models. Direct confirmation of pathway conservation in human chondrocytes and large-animal models is essential for establishing translational confidence.

Nevertheless, the gap between preclinical promise and clinical reality remains considerable. The central controversy, whether piezoelectric output magnitude or long-term biointegration is the key determinant of therapeutic efficacy, is indicative of a deeper tension in biomaterials science that requires resolution. Spontaneous OA models, long-term durability data, and large-animal validation constitute critical research gaps, defining a clear translational roadmap that requires demonstrating output stability beyond 24 weeks *in vivo*, validating cartilage repair in a spontaneous OA large-animal model, and establishing MRI-based non-invasive monitoring of piezoelectric function and repair quality.

The next 5 years will determine whether piezoelectric hydrogels can fulfil their therapeutic potential. The convergence of adaptive materials design, multimodal wireless stimulation, and functional reconstruction endpoints offers a realistic pathway from promising preclinical findings to meaningful clinical impact.

## References

[B1] Alvarez-Lorenzo C. Zarur M. Seijo-Rabina A. Blanco-Fernandez B. Rodríguez-Moldes I. Concheiro A. (2023). Physical stimuli-emitting scaffolds: the role of piezoelectricity in tissue regeneration. Mater Today Bio 22, 100740. 10.1016/j.mtbio.2023.100740 PMC10374602 37521523

[B2] Chen S. Tong X. Huo Y. Liu S. Yin Y. Tan M. L. (2024). Piezoelectric biomaterials inspired by nature for applications in biomedicine and nanotechnology. Adv. Mater 36 (35), e2406192. 10.1002/adma.202406192 39003609

[B3] Das K. K. Basu B. Maiti P. Dubey A. K. (2024). Interplay of piezoelectricity and electrical stimulation in tissue engineering and regenerative medicine. Appl. Mater. Today 39, 102332. 10.1016/j.apmt.2024.102332

[B4] Fan P. Fan H. Wang S. (2024). From emerging modalities to advanced applications of hydrogel piezoelectrics based on chitosan, gelatin and related biological macromolecules: a review. Int. J. Biol. Macromol. 262 (Pt 1), 129691. 10.1016/j.ijbiomac.2024.129691 38272406

[B5] Gao C. Wang X. Wu Z. Zhang X. Geng X. Zhang T. (2026). A piezo-mimetic ionic hydrogel harnessing joint motion for cartilage repair. Adv. Sci. (Weinh) 13, e75611. 10.1002/advs.75611 42107061 PMC13336056

[B6] Han Z. Wang F. Xiong W. Meng C. Yao Y. Cui W. (2025). Precise cell type electrical stimulation therapy Via force-electric hydrogel microspheres for cartilage healing. Adv. Mater 37 (7), e2414555. 10.1002/adma.202414555 39659121

[B7] Li B. Wu A. Zhou M. Wang Y. Hu Z. Su L. (2024). Preparation of high-performance barium titanate composite hydrogels by deep eutectic solvent-assisted frontal polymerization. Mater. (Basel) 17 (13), 3262. 10.3390/ma17133262 PMC11242672 38998343

[B8] Li Y. Chen J. Liu S. Wang Z. Zhang S. Mao C. (2024). Biodegradable piezoelectric polymer for cartilage remodeling. Matter 7 (4), 1631–1643. 10.1016/j.matt.2024.01.034

[B9] Li J. Xie Y. Liu G. Bahatibieke A. Zhao J. Kang J. (2024). Bioelectret materials and their bioelectric effects for tissue repair: a review. ACS Appl. Mater Interfaces 16 (30), 38852–38879. 10.1021/acsami.4c07808 39041365

[B10] Li S. Shan Y. Chen J. Chen X. Shi Z. Zhao L. (2025). 3D printing and biomedical applications of piezoelectric composites: a critical review. Adv. Mater. Technol. 10 (5), 2401160. 10.1002/admt.202401160

[B11] Li X. Liu Y. Yang Q. Zhang W. Wang H. Zhang W. (2025a). Injectable piezoelectric hydrogel promotes tendon-bone healing via reshaping the electrophysiological microenvironment and M2 macrophage polarization. ACS Appl. Mater Interfaces 17 (15), 22210–22231. 10.1021/acsami.4c21011 40178926 PMC12012719

[B12] Li X. Wang F. Zhang X. Liao B. Tan J. He X. (2025b). Ultrasound-responsive piezoelectric analgesic microspheres alleviate osteoarthritis pain. J. Control Release 385, 114049. 10.1016/j.jconrel.2025.114049 40695377

[B13] Lin Z. Nan L. Lu H. Wang Z. Jia C. Zhang Z. (2025). Print-and-Roll hierarchically structured piezoelectric scaffolds for exercise-driven cartilage regeneration. Adv. Funct. Mater. 35 (22), 2424448. 10.1002/adfm.202424448

[B14] Lin J. Li S. Peng Y. Qiu C. Yang M. Guo J. (2025). Ultrasound-triggered injectable piezoelectric nanocomposite hyaluronic acid-based hydrogel for modulating inflammation and chondrogenesis in osteoarthritis treatment. Carbohydr. Polym. 367, 123909. 10.1016/j.carbpol.2025.123909 40817461

[B15] Liu S. Manshaii F. Chen J. Wang X. Wang S. Yin J. (2024). Unleashing the potential of electroactive hybrid biomaterials and self-powered systems for bone therapeutics. Nanomicro Lett. 17 (1), 44. 10.1007/s40820-024-01536-9 39417933 PMC11486894

[B16] Liu X. Wan X. Sui B. Hu Q. Liu Z. Ding T. (2024). Piezoelectric hydrogel for treatment of periodontitis through bioenergetic activation. Bioact. Mater 35, 346–361. 10.1016/j.bioactmat.2024.02.011 38379699 PMC10876489

[B17] Mo G. Qing L. Zhang C. Huang L. Yan H. Lu S. (2026). Ultrasound-activated piezoelectric Silk-PVDF hydrogel reprograms the osteoimmune microenvironment via NRF2 signaling for accelerated bone regeneration. Mater Today Bio 37, 102779. 10.1016/j.mtbio.2026.102779 41660117 PMC12874108

[B18] Ricotti L. Cafarelli A. Manferdini C. Trucco D. Vannozzi L. Gabusi E. (2024). Ultrasound stimulation of piezoelectric nanocomposite hydrogels boosts chondrogenic differentiation *in vitro,*, in both a normal and inflammatory milieu. ACS Nano 18 (3), 2047–2065. 10.1021/acsnano.3c08738 38166155 PMC10811754

[B19] Song Q. Zhang Y. Hu H. Xing X. Wu J. Zhu Y. (2025). Multifunctional hydrogel with synergistic reactive oxygen species scavenging and macrophage polarization-induced osteo-immunomodulation for enhanced bone regeneration. ACS Appl. Mater Interfaces 17 (27), 38985–39001. 10.1021/acsami.5c08737 40552867 PMC12257455

[B20] Tschon M. Codispoti G. Cabras P. Cafarelli A. Trucco D. Vannozzi L. (2026). *In vivo* efficacy of an injectable piezoelectric nanocomposite hydrogel and low-intensity pulsed ultrasound in two preclinical models of osteoarthritis. Biomaterials 326, 123728. 10.1016/j.biomaterials.2025.123728 40997714

[B21] Vannozzi L. Pucci C. Trucco D. Turini C. Sevim S. Pané S. (2025). Biodegradable piezoelectric Micro- and nanomaterials for regenerative medicine, targeted therapy, and microrobotics. Small Sci. 5 (4), 2400439. 10.1002/smsc.202400439 40657196 PMC12245126

[B22] Vijayakanth T. Shankar S. Finkelstein-Zuta G. Rencus-Lazar S. Gilead S. Gazit E. (2023). Perspectives on recent advancements in energy harvesting, sensing and bio-medical applications of piezoelectric gels. Chem. Soc. Rev. 52 (17), 6191–6220. 10.1039/d3cs00202k 37585216 PMC10464879

[B23] Vinikoor T. Dzidotor G. K. Le T. T. Liu Y. Kan H. M. Barui S. (2023). Injectable and biodegradable piezoelectric hydrogel for osteoarthritis treatment. Nat. Commun. 14 (1), 6257. 10.1038/s41467-023-41594-y 37802985 PMC10558537

[B24] Wang H. Xu C. Qin H. He Y. Li Y. Jiang Y. (2026). Piezoelectric nanomotors for active cartilage regeneration of osteoarthritis via ultrasonic vibration and water splitting. Biomaterials 328, 123898. 10.1016/j.biomaterials.2025.123898 41406801

[B25] Wu P. Shen L. Liu H. F. Zou X. H. Zhao J. Huang Y. (2023). The marriage of immunomodulatory, angiogenic, and osteogenic capabilities in a piezoelectric hydrogel tissue engineering scaffold for military medicine. Mil. Med. Res. 10 (1), 35. 10.1186/s40779-023-00469-5 37525300 PMC10388535

[B26] Xu M. Wen Y. Shi Z. Xiong C. Zhu F. Yang Q. (2024). Piezoelectric biopolymers: advancements in energy harvesting and biomedical applications. Polym. (Basel) 16 (23), 3314. 10.3390/polym16233314 39684056 PMC11644150

[B27] Yang S. Wang Y. Liang X. (2023). Piezoelectric nanomaterials activated by ultrasound in disease treatment. Pharmaceutics 15 (5), 1338. 10.3390/pharmaceutics15051338 37242580 PMC10223188

[B28] Yao Y. Gao Z. Pan Z. Zhong Y. Qin L. Zhao K. (2025). Ultrasound-triggered piezocatalytic MOF(Hf)-Pt nanozymes for efficient ROS scavenging in the treatment of osteoarthritis. Chem. Eng. J. 522, 167229. 10.1016/j.cej.2025.167229

[B29] Yue X. Wang Z. Shi H. Wu R. Feng Y. Yuan L. (2023). Silk fibroin-based piezoelectric nanofibrous scaffolds for rapid wound healing. Biomater. Sci. 11 (15), 5232–5239. 10.1039/d3bm00308f 37338183

[B30] Zhang J. Liu C. Li J. Yu T. Ruan J. Yang F. (2025). Advanced piezoelectric materials, devices, and systems for orthopedic medicine. Adv. Sci. (Weinh) 12 (3), e2410400. 10.1002/advs.202410400 39665130 PMC11744659

[B31] Zheng H. Yan P. Liu P. Yan C. Zhao M. Wang Z. (2026). Ultrasound-activatable piezoelectric hydrogel reprograms mitochondrial epigenetics for osteoarthritis therapy via the mTOR/GATD3A axis. Adv. Sci. (Weinh), e76140. 10.1002/advs.76140 42301238 PMC13337106

[B32] Zhou Z. Zheng J. Meng X. Wang F. (2023). Effects of electrical stimulation on articular cartilage regeneration with a focus on piezoelectric biomaterials for articular cartilage tissue repair and engineering. Int. J. Mol. Sci. 24 (3), 1836. 10.3390/ijms24031836 36768157 PMC9915254

